# A Dual-Center Cohort Study on The Association Between Early Deep Sedation and Clinical Outcomes in Mechanically Ventilated Patients During the COVID-19 Pandemic: the COVID-SED Study

**DOI:** 10.21203/rs.3.rs-1389892/v1

**Published:** 2022-03-01

**Authors:** Robert J. Stephens, Erin M. Evans, Michael J. Pajor, Ryan D. Pappal, Haley M. Egan, Max Wei, Hunter Hayes, Jason A. Morris, Nicholas Becker, Brian W. Roberts, Marin H. Kollef, Nicholas M. Mohr, Brian M. Fuller

**Affiliations:** Barnes-Jewish Hospital; University of Iowa; Barnes-Jewish Hospital; Washington University in St. Louis; University of Iowa; University of Iowa; University of Iowa; Harvard Affiliated Emergency Medicine Residency; Mount Sinai Medical Center; Cooper University Hospital; Washington University in St. Louis; University of Iowa; Washington University in St. Louis

**Keywords:** COVID, deep sedation, emergency department, mechanical ventilation

## Abstract

**Background:**

Mechanically ventilated patients have experienced greater periods of prolonged deep sedation during the coronavirus disease (COVID-19) pandemic. Multiple studies from the pre-COVID era demonstrate that early deep sedation is associated with worse outcome. Despite this, there is a lack of data on sedation depth and its impact on outcome for mechanically ventilated patients during the COVID-19 pandemic. We sought to characterize the emergency department (ED) and intensive care unit (ICU) sedation practices during the COVID-19 pandemic, and to determine if early deep sedation was associated with worse clinical outcomes.

**Study Design and Methods:**

Dual-center, retrospective cohort study conducted over six months (March – August, 2020), involving consecutive, mechanically ventilated adults. All sedation-related data during the first 48 hours were collected. Deep sedation was defined as Richmond Agitation-Sedation Scale of −3 to −5 or Riker Sedation-Agitation Scale of 1 – 3. To examine impact of early sedation depth on hospital mortality (primary outcome) we used a multivariable logistic regression model. Secondary outcomes included ventilator-, ICU-, and hospital-free days.

**Results:**

391 patients were studied, and 283 (72.4%) experienced early deep sedation. Deeply sedated patients received higher cumulative doses of fentanyl, propofol, midazolam, and ketamine when compared to light sedation. Deep sedation patients experienced fewer ventilator-, ICU-, and hospital-free days, and greater mortality (30.4% versus 11.1%) when compared to light sedation (*p* < 0.01 for all). After adjusting for confounders, early deep sedation remained significantly associated with higher mortality (adjusted OR 3.44; 95% CI 1.65 – 7.17; p <0.01). These results were stable in the subgroup of patients with COVID-19.

**Conclusions:**

The management of sedation for mechanically ventilated patients in the ICU has changed during the COVID pandemic. Early deep sedation is common and independently associated with worse clinical outcomes. A protocol-driven approach to sedation, targeting light sedation as early as possible, should continue to remain the default approach.

**Clinical Trial Registration:**

Not applicable.

## Introduction

Approximately 95% of all critical care trials have failed to demonstrate benefit (1). Despite this, outcomes for the critically ill have improved over the last several decades, owing not to disease- or syndrome-specific pharmacotherapies, but secondary to improved supportive routine care. Generated from well-designed clinical trials and now guideline-supported, some of these routine care practices include lung-protective ventilation with lower tidal volume, conservative fluid management, the use of checklists, and early mobility (2–5). Sedation management is another critical supportive therapy in mechanically ventilated patients. Specifically, a protocol-driven approach, which favors paired spontaneous awakening (SAT) and breathing (SBT) trials, along with light levels of sedation, improves outcome (6–12). The early period of respiratory failure [i.e. the emergency department (ED) and first 48 hours of intensive care unit (ICU)] may be especially critical to reduce the overall time spent with periods of deep sedation and coma (13–19).

However, there is little rigorous data on sedation depth and its impact on outcome for mechanically ventilated patients during the coronavirus disease (COVID)-19 era. As an example, a PubMed search (conducted on October 7, 2021) for “COVID-19” yielded 184,897 results; “COVID-19 AND sedation” yielded only 287, of which only one cohort study examined the impact of sedation depth on outcome (20, 21). In a comparison of patients with COVID-19-associated acute respiratory distress syndrome (ARDS) with historical ARDS controls, deep sedation and coma were common and associated with increased mortality (20). High rates of delirium and coma have been observed in critically ill patients with COVID-19 infection (22). Concerns have been raised that surges of COVID-19 cases have impacted the care of critically ill patients without COVID-19 disease, potentially worsening outcomes(23). Overall, these findings suggest that the impact of early deep sedation on outcome during the COVID-19 pandemic, for patients with and without COVID-19, is incompletely understood.

We therefore conducted the COVID-SED Study to: 1) further characterize ED and ICU sedation practices during the COVID-19 pandemic; and 2) test the hypothesis that early deep sedation is associated with worse clinical outcomes.

## Methods

### Study Design

This is a retrospective cohort study conducted over six months (March – August, 2020), involving consecutive adult mechanically ventilated patients at two academic tertiary referral centers. The study is reported in accordance with the Strengthening Reporting of Observational Studies in Epidemiology (STROBE) Statement. (Additional File 1) (24).

The Institutional Review Board (IRB) and Human Research Protection Office (HRPO) at each site approved the study with waiver of informed consent prior to study initiation (IRB # 202009119 and 202009604).

### Participants

All consecutive mechanically ventilated adult patients admitted to the ICU from the ED were screened via established electronic screening procedures. Inclusion criterion: 1) age ≥ 18 years; and 2) receipt of mechanical ventilation via an endotracheal tube. In addition to mechanically ventilated patients admitted from the ED, all other mechanically ventilated COVID-19 patients admitted to the intensive care unit were screened for inclusion. This was done to capture all patients with COVID-19 during the six-month enrollment period, provided they satisfied all other inclusion and exclusion criteria. Exclusion criteria targeted patients in whom duration of mechanical ventilation was unlikely to be altered by sedation management or those in whom acute injury could act as a confounder with sedation depth: 1) death or transition to comfort measures within 24 hours; 2) acute neurologic injury (e.g. stroke, intracranial hemorrhage, traumatic brain injury, cardiac arrest with residual neurologic deficit, status epilepticus, drug overdose, fulminant hepatic failure); 3) transfer to another hospital; 4) chronic/home ventilation; 5) direct admission to the operating room (OR) from the ED; and 6) extubation in the ED.

### Assessments and Outcome Measures

Clinical variables and outcome measures were objective to ensure ease of abstraction from the electronic medical record. Data were collected and entered into a database with Research Electronic Data Capture (REDCap) tools (25, 26). Team members were trained regarding data abstraction. Data quality checks were performed with manual and automated methods, and by enforcing plausible data ranges in the REDCap fields. Prior to analysis, the database was screened for implausible values and the electronic medical record was used to recheck any flagged data.

Baseline data including age, gender, weight, race, comorbid medical conditions, COVID-19 status, vital signs, laboratory values, indication for mechanical ventilation, and ventilator settings were recorded. Process of care variables included ED length of stay, antibiotic use, and vasopressor use. Illness severity was assessed with the modified sequential organ failure assessment (SOFA) score (27, 28).

Sedation-related data included induction agents and neuromuscular blockers used for endotracheal intubation. Analgesia- and sedation-related data from the ED and during the first 48 hours of ICU admission included opiates, propofol, benzodiazepines, dexmedetomidine, ketamine, haloperidol, quetiapine, gabapentin, and neuromuscular blockers (i.e. rocuronium, vecuronium, and cisatricurium).

Sedation depth was monitored and recorded according to standard routine care at each site, and included the Richmond Agitation-Sedation Scale (RASS) and the Riker Sedation-Agitation Scale (SAS). Deep sedation was defined as: 1) median RASS of −3 to −5; or 2) median SAS of 1–3 (15–17, 29) during the first 48 hours of care from admission to the ICU. This period of early sedation was chosen for several reasons. First, early sedation depth is appears to be an important contributor to outcome in mechanically ventilated patients, as demonstrated by several studies which found deep sedation during the initial 48 hours of mechanical ventilation to be associated with increased mechanical ventilation duration, mortality, incidence of delirium, and longer lengths of stay (14–16, 19). Second, this endpoint would allow for an account of the time spent in the ED, which has not been reported before during the COVID-19 pandemic.

Patients were followed until death or hospital discharge. The primary outcome was hospital mortality. Secondary outcomes include ventilator-, ICU-, and hospital-free days.

### Statistical Analysis

Descriptive statistics and frequency distributions were used to assess baseline patient characteristics and sedation-related data according to sedation depth. Categorical data were compared with the chi-square test, and continuous data were compared using the independent samples t-test or Mann-Whitney U test after testing for normality of data. Time (in days) to mortality was assessed with the Kaplan-Meier survival estimate and log-rank test, comparing the early deep sedation and light sedation groups. A second Kaplan-Meier survival estimate was also calculated, which also included patients deeply sedated throughout the first week of ICU care.

To examine the impact of early sedation depth on hospital mortality, a multivariable logistic regression model was used, following recommendations that covariates be selected *a priori* (30). The model was adjusted for covariates previously associated with mortality in this cohort: 1) early deep sedation; 2) age; 3) illness severity; 4) indication for mechanical ventilation; and 5) COVID-19 status. All tests were two-tailed and a *p* value of < 0.05 was considered statistically significant.

A *post-hoc* exploratory analysis was conducted after noting a significantly higher proportion of deeply sedated COVID-19 patients (Table 1). Taking a similar approach to the primary analysis, this secondary analysis analyzed and reported the baseline characteristics and sedation-related data according to COVID-19 status. To further explore if deep sedation remained independently associated with worse clinical outcomes, a separate multivariable model was conducted on patients positive for COVID-19.

From prior work regarding the impact of early deep sedation on outcome, we estimated that approximately two-thirds of the cohort would experience early deep sedation, with a mortality of 25% in the early deep sedation group versus 10% in the light sedation group (19). For 80% power and alpha of 0.05, we estimated a sample size of 219 (82 light sedation, 137 deep sedation) would be required. Based on our prior work regarding mechanically ventilated patients at each site, we were confident that a six-month enrollment window would be sufficient to accrue the necessary sample size (17, 18, 31–34).

## Results

The data presented here was from the first six months of the COVID-19 pandemic and we recognize that practices have evolved dramatically since March of 2020.

### Study Population

**Figure 1:**
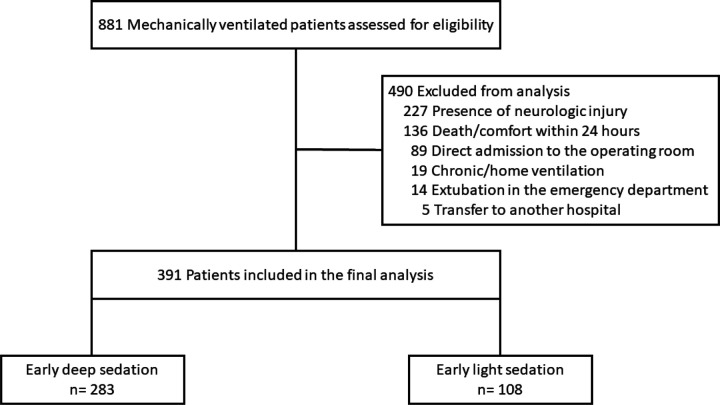
Study flow diagram

Eight hundred eighty-one patients were assessed for eligibility, and 391 comprised the final study population (Fig. 1). Baseline characteristics according to early sedation depth status are in Table 1. Deeply sedated patients had a higher proportion of patients with COVID-19, and a lower partial pressure of arterial oxygenation to fraction of inspired oxygen ratio (PaO2:FiO2).

### Medications Administered

Medications used for endotracheal intubation are located in Additional Table 1. Sedation variables for the 244 patients that were mechanically ventilated in the ED are in Additional Table 2. ICU sedation variables for the first 48 hours of admission are in Table 2. Deeply sedated patients received higher cumulative doses of fentanyl, propofol, midazolam, hydromorphone, and ketamine when compared to the light sedation group. In addition, deeply sedated patients received neuromuscular blockers more frequently (41.4% vs. 2.1%, *p* < 0.01).

### Depth of Sedation

Deep sedation occurred in 72.4% of all patients (both COVID-19 and non-COVID-19 cohorts) during the first 48 hours. Sedation levels differed significantly (*p* < 0.01 for each) between the deep sedation and light sedation groups during this period. This difference persisted through the first seven days of mechanical ventilation (Table 2), such that 128 (53.8%) patients in the deep sedation group experienced deep sedation during the first week of ICU care, as compared to 14 (18.4%) patients in the light sedation group, *p <* 0.01. Ninety-four (33.2%) deeply sedated patients remained deeply sedated until death, compared to 0 (0.0%) patients in the light sedation group, *p* < 0.01.

### Subgroup Analyses

Baseline characteristics according to COVID-19 status are in Additional Table 3. ED sedation variables are in Additional Table 4, and ICU sedation variables from the first 48 hours are in Table 3. No significant differences in medication doses were observed in the ED. In the ICU, COVID-19 patients received significantly higher cumulative doses of fentanyl, propofol, midazolam, hydromorphone, and ketamine when compared to non-COVID patients. COVID-19 patients also received neuromuscular blockers more frequently than non-COVID patients in the ICU (41.4% vs. 2.1%, *p* < 0.01). COVID-19 patients experienced deep sedation more frequently early and throughout the first week of ICU care (*p* < 0.01 for all). Seventy-eight (38.4%) COVID patients remained deeply sedated until death, compared to 16 (8.5%) non-COVID patients.

### Clinical Outcomes

**Figure 2:**
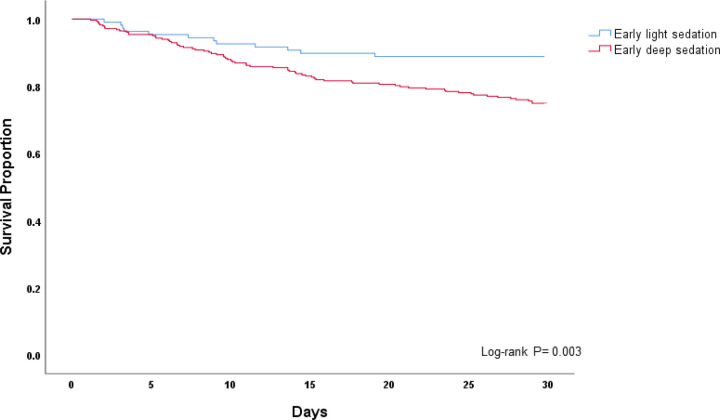
Kaplan-Meier survival curve between the early deep and light sedation groups

Table 4 shows that in the unadjusted analysis of clinical outcomes according to sedation depth, deep sedation patients experienced fewer ventilator-, ICU-, and hospital-free days, and greater mortality (30.4% versus 11.1%) when compared to light sedation (*p* < 0.01 for all). On Kaplan-Meier analysis, survival diverged significantly between the early deep sedation and light sedation groups (log-rank p < 0.01, Fig. 2). After adjusting for confounders (Additional Table 5), early deep sedation remained significantly associated with higher mortality (adjusted OR 3.44; 95% CI 1.65–7.17; p < 0.01).

In the subgroup analysis (Additional Table 6), similar unadjusted clinical outcomes according to COVID status were seen, such that COVID patients experienced fewer ventilator-, ICU-, and hospital-free days (*p* < 0.01 for all). Mortality was 41.4% in COVID patients versus 7.4% in non-COVID patients (*p* < 0.01). After adjusting for confounders (Additional Table 6), early deep sedation remained significantly associated with higher mortality (adjusted OR 2.76; 95% CI 1.26–6.06; p < 0.01), though illness severity remained an important variable in this analysis.

## Discussion

Given the importance of high-quality supportive therapies in critical illness, the potential impact of early sedation depth on clinical outcomes, and a dearth of early sedation data in the COVID-19 era, we conducted the COVID-SED study to characterize ED and early ICU sedation practices during the COVID-19 pandemic and assess the impact of early deep sedation on clinical outcomes. We found that over 70% of mechanically ventilated patients experienced early deep sedation, with significant differences in cumulative medication doses and neuromuscular blockade. In addition, early deep sedation frequently persisted throughout the first week of mechanical ventilation and was negatively associated with outcome.

Our most important finding was an association between early deep sedation and worse clinical outcomes. Early deep sedation was associated with fewer ventilator-, ICU-, and hospital-free days, and increased hospital mortality. These results remained significant after adjustment for confounders, and were consistent in the subgroup of patients with COVID. Our findings are supported by prior work in the pre-COVID era, which showed the negative relationship between early deep sedation and patient-centered clinical outcomes (14–19). Additionally, these findings are congruent with a recent analysis that examined the impact of deep sedation in a comparison of patients with COVID-associated ARDS with non-COVID historical controls (20). The findings of the COVID-SED Study are further support of a guideline- and protocol-driven approach to sedation management, regardless of COVID status (35).

A second important finding is the characterization of sedation practices during the first wave of the COVID pandemic. Sedation in the ED was similar to prior work, suggesting that the COVID era influenced ED-based sedation little (17). However, compared with pre-COVID work, sedation in the ICU saw an increased use and higher doses of fentanyl, benzodiazepines, and ketamine, which appeared largely driven by COVID status (17). The occurrence rate of 72.4% of early deep sedation is also higher than that seen in recent pre-COVID publications and further highlights the rapidly-adopted changes in sedation practice that occurred with the COVID pandemic (17, 19). These findings are consistent with prior reports that documented high sedative and neuromuscular blockade use in COVID patients(20, 22, 36–39). Further, our findings highlight the static nature in the approach to sedation in the early deep sedation group: 1) > 50% experienced deep sedation throughout the first week of mechanical ventilation; and 2) 33% were deeply sedated until death. While not formally measured in this study, these results further suggest low adherence to the ABCDEF bundle, congruent with a prior international point prevalence study on ICU patients with COVID (40).

Another important finding involves the sedation observed in non-COVID patients. Given the significant changes in supportive care observed during the onset of the COVID pandemic, it is reasonable to hypothesize that the care of non-COVID patients would have been altered as well. However, when compared to prior work, patients in the non-COVID group experienced sedation management, early deep sedation, and clinical outcomes similar to that seen in the pre-COVID era (17). This suggests that the observed changes in the standards of critical care were isolated to COVID patients, and further highlights the importance of continued assessments into protocol-driven supportive care in this cohort.

This work has several important limitations. This is one of the first studies examining the impact of sedation depth on clinical outcomes during the COVID pandemic, yet it is relatively small and therefore prone to bias. As a two-center study, it is possible that these data are not truly representative and lack external validity. All data were obtained retrospectively and therefore subject to potential inaccuracies in routine documentation. The study design can only inform on association and not causation, and the ability to control for confounding is limited. Deep sedation, and therefore the possible the need for it, overlapped with COVID status, and may also have been a marker of illness severity and the presence of ARDS. Our results are consistent with prior literature regarding the impact of early deep sedation on outcomes, and the association between deep sedation and mortality remained strong after adjusting for SOFA (which includes oxygenation). While this is encouraging and lends face validity, however the relationship between early deep sedation and disease severity is difficult to truly separate through statistical methods. As such, these results should be viewed as hypothesis-generating. These data were collected during the first six months of the COVID pandemic, and therefore may not reflect rapidly evolving COVID era sedation practices. However, this work highlights the importance of adhering to proven ICU principles and are informative for the potential of persistent COVID-19 or future viral pandemics. Finally, depressed mental status and deeper sedation levels may have been secondary to COVID and/or structural lesions, as opposed to sedation management (41). Since no imaging data were collected for this study, this remains a potential confounder.

## Conclusion

The management of sedation for mechanically ventilated patients in the ICU has been impacted by the COVID pandemic. Early deep sedation is common, especially among COVID-19 patients, and independently associated with worse clinical outcomes. A protocol-driven approach to sedation, targeting light sedation as early as possible, should continue to remain the default approach.

## Supplementary Material

Supplement 2

Supplement 3

Supplement 4

Supplement 5

Supplement 6

Supplement 7

Supplement 1

## Figures and Tables

**Table 1:** Characteristics of mechanically ventilated patients based on early sedation depth status.

Early Sedation Depth Status			

Baseline characteristics	Light Sedation (n = 108)	Deep Sedation (n = 283)	P value

Age (yr)	55.2 (19.4)	56.4 (16.6)	0.53

Gender	65 (60.2)	169 (59.7)	0.93
Male, n (%)	43 (39.8)	114 (40.3)	
Female, n (%)			

Body mass index (kg/m^2^)	29.5 (8.6)	30.0 (9.6)	0.61

Race, n (%)	42 (38.9)	138 (48.8)	0.48
White	58 (53.7)	120 (42.4)	
Black	3 (2.8)	10 (3.5)	
Hispanic	1 (0.9)	4 (1.4)	
Asian	0 (0.0)	1 (0.3)	
Native American	4 (3.7)	10 (3.5)	
Other			
Comorbidities, n (%)	8 (7.4)	28 (9.9)	0.45
Dementia	30 (27.8)	106 (37.5)	0.07
Diabetes mellitus	6 (5.6)	13 (4.6)	0.69
Cirrhosis	15 (13.9)	51 (18.0)	0.33
CHF	9 (8.3)	20 (7.1)	0.67
ESRD/Dialysis	18 (16.7)	52 (18.4)	0.69
COPD	4 (3.7)	18 (6.4)	0.31
Immunosuppression	11 (10.2)	36 (12.7)	0.49
Malignancy	16 (14.8)	27 (9.5)	0.14
Alcohol abuse	37 (34.3)	83 (29.3)	0.35
Psychiatric*			

Positive for COVID-19	44 (40.7)	159 (56.2)	0.01

Temperature (Celsius)	36.9 (1.3)	37.0 (1.4)	0.31

Blood pressure (mmHg)	132.7 (34.4)	128.0 (29.8)	0.19
Systolic	82.0 (24.2)	79.5 (21.6)	0.33
Diastolic			

Lactate (mmol/L)	2.0 (1.3–3.1)	2.1 (1.3–3.4)	0.61

Creatinine (mg/dl)	1.1 (0.8–1.8)	1.2 (0.9–2.3)	0.10

Hemoglobin (g/dl)	12.4 (2.5)	12.4 (2.5)	0.85

pH	7.30 (0.12)	7.30 (0.12)	0.95

PaO2	137.0 (70.9)	121.0 (76.9)	0.23

PaO2:FiO2	241.3 (161.0)	184.8 (148.3)	0.04

PaCO2	49.4 (16.9)	48.7 (19.9)	0.77

SOFA**	4.5 (2.6)	5.3 (2.5)	0.01

Reason for mechanical ventilation, n (%)	14 (13.0)	44 (15.5)	0.01
Sepsis	18 (16.7)	23 (8.1)	
Trauma	17 (15.7)	48 (17.0)	
COPD	12 (11.1)	12 (4.2)	
Drug overdose	10 (9.3)	22 (7.8)	
CHF/pulmonary edema	13 (12.0)	76 (26.9)	
Other	4 (3.7)	10 (3.5)	
Cardiac arrest	10 (9.3)	21 (7.4)	
Altered mental status	1 (0.9)	5 (1.8)	
Angioedema	1 (0.5)	0 (0.0)	
Neuromuscular weakness	8 (7.4)	22 (7.8)	
Airway protection			

Tidal volume (mL/kg PBW)	6.6 (6.1–7.3)	6.5 (6.0–7.3)	0.24

PEEP (cm H20)	6.5 (5.0–10.0)	8.0 (5.0–12.0)	< 0.01

Fraction of inspired oxygen (%)	64.8 (25.9)	74.9 (26.2)	< 0.01

**Process of Care Variables**			

ED length of stay (hours)	5.9 (3.8–8.3)	4.0 (2.5–6.1)	< 0.01

Antibiotics for infection, n (%)	50 (47.6)	119 (44.2)	0.56

Vasopressor infusion, n (%)	26 (24.3)	72 (25.9)	0.75

CHF: congestive heart failure; ESRD: end-stage renal disease; COPD: chronic obstructive pulmonary disease; SOFA: sequential organ failure assessment score; PEEP: positive end-expiratory pressure; ED: emergency department

Continuous variables are reported as mean (standard deviation) and median (interquartile range).

* schizophrenia, bipolar disorder, major depression, anxiety

** modified score, which excludes Glasgow Coma Scale

**Table 2:** Sedation variables in the intensive care unit during the first 48 hours of admission, according to sedation depth

Early Sedation Depth Status			

Drug	Light Sedation (n = 108)	Deep Sedation (n = 283)	p

Fentanyl	92 (85.2)	240 (84.8)	0.93
n (%)	3175 (1206–6330)	3950 (1600–6950)	0.09
Cumulative dose (mcg)			

Propofol	85 (78.7)	222 (78.4)	0.96
n (%)	1526 (600–4914)	4047 (1507–8109)	< 0.01
Cumulative dose (mg)			

Midazolam	35 (32.4)	115 (40.6)	0.14
n (%)	12.0 (3.0–54.0)	19.0 (5.0–152.0)	0.09
Cumulative dose (mg)			

Dexmedetomidine	50 (46.3)	93 (32.9)	0.01
n (%)	4.6 (2.0–9.5)	7.0 (2.1–17.8)	0.08
Cumulative dose (mcg/kg)			

Lorazepam	11 (10.2)	27 (9.5)	0.85
n (%)	3.0 (1.0–12.0)	2.0 (1.0–3.0)	0.32
Cumulative dose (mg)			

Hydromorphone	12 (11.1)	49 (17.3)	0.13
n (%)	2.5 (1.0–17.8)	9.0 (3.0–69.0)	0.04
Cumulative dose (mg)			

Oxycodone	18 (16.7)	28 (9.9)	0.06
n (%)	17.5 (10.0–32.5)	20.0 (10.0–40.0)	0.96
Cumulative dose (mg)			

Morphine	1 (0.9)	7 (2.5)	0.33
n (%)	2.0 (NA)	6.5 (2.0–12.8)	0.57
Cumulative dose (mg)			

Ketamine	10 (9.3)	38 (13.4)	0.26
n (%)	87.5 (50.0–250.0)	675.0 (187.5–2050.0)	< 0.01
Cumulative dose (mg)			

Haloperidol	9 (8.3)	13 (4.6)	0.15
n (%)	5.0 (5.0–10.0)	5.0 (5.0–10.0)	0.95
Cumulative dose (mg)			

Quetiapine	4 (3.7)	12 (4.2)	0.81
n (%)	37.5 (25.0–237.5)	200.0 (31.3–287.5)	0.91
Cumulative dose (mg)			

Gabapentin	11 (10.2)	11 (3.9)	0.02
n (%)	600.0 (300.0–2100.0)	1200 (300–2100)	0.33
Cumulative dose (mg)			

Neuromuscular blocker, n (%)	4 (2.1)	84 (41.4)	< 0.01

RASS Level ICU Day 1	−1 (−2 to −0)	−3 (−4 to −2)	< 0.01
SAS Level ICU Day 1	4 (4–4)	3 (2–4)	< 0.01

RASS Level ICU Day 2	−1 (−2 to 0)	−3 (−5 to −2)	< 0.01
SAS Level ICU Day 2	4 (4–4)	3 (3–4)	< 0.01

RASS Level ICU Days 3–7	−1 (−2 to 0)	−3 (−4 to −1)	< 0.01
SAS Level ICU Days 3–7	4 (4–4)	3 (3–4)	< 0.01

Deep sedation ICU Days 3–7, n (%)*	14 (18.4)	128 (53.8)	< 0.01
Deep sedation until death, n (%)	0 (0.0)	94 (33.2)	< 0.01

ICU = intensive care unit, RASS = Richmond Agitation-Sedation Scale.

* Denominator is 314 (238 deep sedation group and 76 light sedation group).

**Table 3:** Sedation variables in the intensive care unit during the first 48 hours of admission, according to COVID status.

COVID Status			

Drug	Non-COVID (n = 188)	COVID (n = 203)	p

Fentanyl	148 (78.7)	184 (90.6)	< 0.01
n (%)	1562 (509–4063)	5350 (3275–8050)	< 0.01
Cumulative dose (mcg)			

Propofol	143 (76.1)	164 (80.8)	0.26
n (%)	2324 (1021–6443)	4047 (1227–8127)	0.02
Cumulative dose (mg)			

Midazolam	36 (19.1)	114 (56.2)	< 0.01
n (%)	4.0 (2.0–30.0)	31.5 (5.0–155.0)	< 0.01
Cumulative dose (mg)			

Dexmedetomidine	91 (48.4)	52 (25.6)	< 0.01
n (%)	5.3 (2.2–15.4)	5.3 (1.6–13.1)	0.67
Cumulative dose (mcg/kg)			

Lorazepam	17 (9.0)	21 (10.3)	0.66
n (%)	2.0 (1.5–11.0)	2.0 (1.0–3.5)	0.37
Cumulative dose (mg)			

Hydromorphone	38 (20.2)	23 (11.3)	0.02
n (%)	4.5 (2.0–11.0)	71.0 (9.0–108.0)	< 0.01
Cumulative dose (mg)			

Oxycodone	28 (14.9)	18 (8.9)	0.07
n (%)	17.5 (10.0–37.5)	20.0 (10.0–35.0)	0.76
Cumulative dose (mg)			

Morphine	1 (0.5)	7 (3.4)	0.04
n (%)	8.0 (NA)	3.5 (2.0–12.8)	0.86
Cumulative dose (mg)			

Ketamine	16 (8.5)	32 (15.8)	0.03
n (%)	92.5 (50.0–350.0)	950.0 (234.0–2050.0)	< 0.01
Cumulative dose (mg)			

Haloperidol	14 (7.4)	8 (3.9)	0.13
n (%)	5.0 (5.0–11.3)	5.0 (5.0–8.8)	0.37
Cumulative dose (mg)			

Quetiapine	5 (2.7)	11 (5.4)	0.17
n (%)	50.0 (37.5–300.0)	200.0 (25.0–250.0)	0.91
Cumulative dose (mg)			

Gabapentin	13 (6.9)	9 (4.4)	0.29
n (%)	600.0 (300.0–2100.0)	800.0 (350.0–2100.0)	0.85
Cumulative dose (mg)			

Neuromuscular blocker	4 (2.1)	84 (41.4)	< 0.01
n (%)			

RASS Level ICU Day 1	−2 (−3 to −1)	−3 (−4 to −2)	< 0.01
SAS Level ICU Day 1	3 (3–4)	2 (1–4)	< 0.01

RASS Level ICU Day 2	−1 (−2 to 0)	−3 (−5 to −2)	< 0.01
SAS Level ICU Day 2	4 (3–4)	3 (1–3)	< 0.01

RASS Level ICU Days 3–7	0 (−2 to 0)	−3 (−5 to −2)	< 0.01
SAS Level ICU Days 3–7	4 (3–4)	2 (1–3)	< 0.01

Deep sedation ICU Day 1, n (%)	73 (38.8)	118 (58.1)	< 0.01
Deep sedation ICU Day 2, n (%)*	43 (25.9)	117 (60.6)	< 0.01
Deep sedation ICU Days 3–7, n (%)**	25 (19.4)	109 (61.6)	< 0.01
Deep sedation until death, n (%)	16 (8.5)	78 (38.4)	< 0.01

ICU = intensive care unit, RASS = Richmond Agitation-Sedation Scale.

* Denominator is 359 (193 COVID group and 166 non-COVID group).

** Denominator is 314 (185 COVID group and 129 non-COVID group).

**Table 4:** Unadjusted analysis of clinical outcomes according to early sedation depth.

Outcome	Light sedation (n = 108)	Deep sedation (n = 283)	OR or Between-Group Difference (95% CI)	p
Ventilator-free days	20.7 (9.6)	14.7 (11.4)	6.04 (3.60–8.48)	< 0.01
ICU-free days	18.3 (9.9)	12.1 (11.0)	6.20 (3.82–8.57)	< 0.01
Hospital-free days	13.8 (10.3)	8.0 (9.6)	5.74 (3.56–7.92)	< 0.01
Mortality, n (%)	12 (11.1)	86 (30.4)	3.49 (1.82–6.70)	< 0.01

ICU = intensive care unit; OR: odds ratio; CI: confidence interval

## Abbreviations

ARDS: acute respiratory distress syndrome
COVID: coronavirus disease
ED: emergency department
FiO2: fraction of inspired oxygen
HRPO: Human Research Protection Office
ICU: intensive care unit
IRB: Institutional Review Board
OR: operating room
PaO2: partial pressure of arterial oxygenation
RASS: Richmond Agitation-Sedation Scale
SAS: Sedation-Agitation Scale
SOFA: sequential organ failure assessment
STROBE: Strengthening Reporting of Observational Studies in Epidemiology
