# Supplementary material for: A Dual-Center Cohort Study on The Association Between Early Deep Sedation and Clinical Outcomes in Mechanically Ventilated Patients During the COVID-19 Pandemic: the COVID-SED Study
Source: Res Sq. 2022 Mar 1:rs.3.rs-1389892. Preprint. [Version 1] doi: 10.21203/rs.3.rs-1389892/v1 (PMC8902881; doi:10.21203/rs.3.rs-1389892/v1)
Supplement: Supplement 3 [file f08ba36cf1f2d367e915024c.docx]

**Additional Table 2.** Sedation variables, according to sedation depth, for the 244 patients that received mechanical ventilation in the emergency department.

| **Early Sedation Depth Status** | | | |
| --- | --- | --- | --- |
| **Drug** | **Light Sedation**  **(n= 83)** | **Deep Sedation**  **(n= 161)** | ***p*** |
| Fentanyl  n (%)  Cumulative dose (mcg) | 69 (83.1)  350 (175.0 – 800.0) | 114 (70.8)  200.0 (100.0 – 400.0) | 0.04  <0.01 |
| Propofol  n (%)  Cumulative dose (mg) | 61 (73.5)  426.7 (168.5 – 783.6) | 99 (61.5)  344.5 (189.9 – 813.1) | 0.06  0.77 |
| Midazolam  n (%)  Cumulative dose (mg) | 20 (24.1)  6.0 (4.0 – 10.8) | 37 (23.0)  5.0 (2.0 – 9.0) | 0.85  0.21 |
| Dexmedetomidine  n (%)  Cumulative dose (mcg) | 6 (7.2)  1.3 (0.48 – 2.3) | 12 (7.5)  1.7 (1.1 – 2.4) | 0.95  0.44 |
| Ketamine  n (%)  Cumulative dose (mg) | 11 (13.3)  100.0 (50.0 – 200.0) | 18 (11.2)  112.5 (92.5 – 200.0) | 0.64  0.41 |
| Lorazepam  n (%)  Cumulative dose (mg) | 9 (10.8)  2.0 (1.5 – 3.5) | 18 (11.2)  3.0 (1.0 – 4.0) | 0.94  0.71 |
| Hydromorphone  n (%)  Cumulative dose (mg) | 4 (4.8)  1.0 (1.0 – 1.8) | 3 (1.9)  1.0 (NA) | 0.19  1.0 |
| Haloperidol  n (%)  Cumulative dose (mg) | 4 (4.8)  7.5 (5.0 – 11.5) | 2 (1.2)  6.5 (5.0 – NA) | 0.09  0.80 |
| Neuromuscular blocker, n (%) | 1 (1.2) | 7 (4.3) | 0.19 |
