# Supplementary material for: A Dual-Center Cohort Study on The Association Between Early Deep Sedation and Clinical Outcomes in Mechanically Ventilated Patients During the COVID-19 Pandemic: the COVID-SED Study
Source: Res Sq. 2022 Mar 1:rs.3.rs-1389892. Preprint. [Version 1] doi: 10.21203/rs.3.rs-1389892/v1 (PMC8902881; doi:10.21203/rs.3.rs-1389892/v1)
Supplement: Supplement 4 [file ce65be694fb4f243fff45aa6.doc]

**Additional Table 3. Characteristics of mechanically ventilated patients based on presence of coronavirus disease.**

| **COVID Status** | | | |
| --- | --- | --- | --- |
| **Baseline characteristics** | **Non-COVID**  **(n= 188)** | **COVID**  **(n= 203)** | ***P* value** |
| Age (yr) | 50.9 (18.4) | 60.8 (15.0) | <0.01 |
| Gender  Male, n (%)  Female, n (%) | 111 (59.0)  77 (41.0) | 123 (60.6)  80 (39.4) | 0.76 |
| Body mass index (kg/m^2^) | 28.0 (8.7) | 31.6 (9.5) | <0.01 |
| Race, n (%)  White  Black  Hispanic  Asian  Native American  Other | 106 (56.9)  69 (36.7)  5 (2.7)  1 (0.5)  1 (0.5)  6 (3.2) | 74 (36.5)  109 (53.7)  8 (3.9)  4 (2.0)  0 (0.0)  8 (3.9) | <0.01 |
| Comorbidities, n (%)  Dementia  Diabetes mellitus  Cirrhosis  CHF  ESRD/Dialysis  COPD  Immunosuppression  Malignancy  Alcohol abuse  Psychiatric* | 12 (6.4)  49 (26.1)  9 (4.8)  38 (20.2)  12 (6.4)  37 (19.7)  9 (4.8)  20 (10.6)  36 (19.1)  70 (37.2) | 24 (11.8)  87 (42.9)  10 (4.9)  28 (13.8)  17 (8.4)  33 (16.3)  13 (6.4)  27 (13.3)  7 (3.4)  50 (24.6) | 0.06  <0.01  0.95  0.09  0.45  0.38  0.49  0.42  <0.01  <0.01 |
| Temperature (Celsius) | 36.6 (1.2) | 37.4 (1.4) | <0.01 |
| Blood pressure (mmHg)  Systolic  Diastolic | 132.0 (32.6)  85.2 (22.5) | 126.8 (29.6)  75.6 (21.1) | 0.10  <0.01 |
| Lactate (mmol/L) | 2.4 (1.4 – 3.6) | 1.9 (1.2 – 2.8) | 0.01 |
| Creatinine (mg/dl) | 1.0 (0.8 – 1.6) | 1.4 (1.0 – 2.5) | <0.01 |
| Hemoglobin (g/dl) | 12.8 (2.2) | 12.1 (2.7) | <0.01 |
| pH | 7.27 (0.12) | 7.32 (0.12 | <0.01 |
| PaO2 | 126.0 (79.0) | 125.3 (72.8) | 0.95 |
| PaO2:FiO2 | 240.1 (176.7) | 169.7 (124.8) | <0.01 |
| PaCO2 | 53.3 (21.3) | 44.0 (14.8) | <0.01 |
| SOFA** | 4.5 (2.4) | 5.6 (2.5) | <0.01 |
| Reason for mechanical ventilation, n (%)  Sepsis  Trauma  COPD  Drug overdose  CHF/pulmonary edema  Other  Cardiac arrest  Altered mental status  Angioedema  Neuromuscular weakness  Airway protection | 14 (7.4)  38 (20.2)  9 (4.8)  22 (11.7)  12 (6.4)  24 (12.8)  10 (5.3)  27 (14.4)  6 (3.2)  1 (0.5)  25 (13.3) | 44 (21.7)  3 (1.5)  56 (27.6)  2 (1.0)  20 (9.9)  65 (32.0)  4 (2.0)  4 (2.0)  0 (0.0)  0 (0.0)  5 (2.5) | <0.01 |
| Tidal volume (mL/kg PBW) | 6.4 (5.8 – 7.3) | 6.6 (6.1 – 7.3) | 0.08 |
| PEEP (cm H20) | 5.0 (5.0 – 8.0) | 10.0 (5.0 – 13.0) | <0.01 |
| Fraction of inspired oxygen (%) | 83.7 (21.9) | 59.7 (25.4) | <0.01 |
| **Process of Care Variables** |  |  |  |
| ED length of stay (hours)  ED mechanical ventilation (hours) | 4.7 (2.8 – 7.5) | 3.9 (3.1 – 5.8) | 0.24 |
| Antibiotics for infection, n (%) | 77 (42.1) | 92 (48.2) | 0.24 |
| Vasopressor infusion, n (%) | 61 (32.4) | 37 (18.8) | <0.01 |

CHF: congestive heart failure; ESRD: end-stage renal disease; COPD: chronic obstructive pulmonary disease; SOFA: sequential organ failure assessment score; PEEP: positive end-expiratory pressure; ED: emergency department

Continuous variables are reported as mean (standard deviation) and median (interquartile range).

* schizophrenia, bipolar disorder, major depression, anxiety

**modified score, which excludes Glasgow Coma Scale
