# Supplementary material for: A Dual-Center Cohort Study on The Association Between Early Deep Sedation and Clinical Outcomes in Mechanically Ventilated Patients During the COVID-19 Pandemic: the COVID-SED Study
Source: Res Sq. 2022 Mar 1:rs.3.rs-1389892. Preprint. [Version 1] doi: 10.21203/rs.3.rs-1389892/v1 (PMC8902881; doi:10.21203/rs.3.rs-1389892/v1)
Supplement: Supplement 6 [file fb1186ed7ec568e6b7f53e01.docx]

**Additional Table 5.** Results of the multivariable logistic regression analysis for the primary outcome of mortality.

| **Variable** | **aOR** | **95% CI** | ***P* value** |
| --- | --- | --- | --- |
| Early deep sedation | 3.44 | 1.65 – 7.17 | <0.01 |
| Age | 1.03 | 1.01 – 1.05 | <0.01 |
| Positive for COVID-19 | 6.43 | 3.39 – 12.19 | <0.01 |
| Total SOFA Score | 1.20 | 1.08 – 1.34 | 0.01 |
| Indication for mechanical ventilation | 1.36 | 0.49 – 3.76 | 0.55 |

SOFA: sequential organ failure assessment; aOR: adjusted odds ration; CI: confidence interval
